# How to Preserve Health

**Published:** 1890-08

**Authors:** 


HOW TO PRESERVE HEALTH. By Louis Barkan, M. D., Supplied by American Medical Company, N. Y., 1890.

A valuable book, in pamphlet form, of three hundred and odd pages, giving in detail many important principles of private and public hygiene. The chapter of hygiene of the different organs of the body will be found instructive and beneficial. Hygiene of the dwelling is a subject every one should be informed about, and in this little work much is to be learned on this subject. ROBT. W. W.

